# The role of pelvic floor muscle training in the management of idiopathic fecal incontinence: a single-center prospective study

**DOI:** 10.1007/s10151-026-03390-w

**Published:** 2026-07-10

**Authors:** Pamela Jeske, Cezary Miedziarek, Dominika Clarke, Maciej Borejsza-Wysocki, Tomasz Banasiewicz, Krzysztof Szmyt

**Affiliations:** 1 Department of General, Endocrinological Surgery and Gastroenterological Oncology, Poznan University of Medical Sciences, Poznan, Poland; 2 Department of Gynaecology, Division of Gynaecological Oncology, Poznan University of Medical Sciences, Przybyszewskiego Street 49, 60-355 Poznan, Poland

**Keywords:** Wexner scale, Physiotherapy, Fecal incontinence

## Abstract

**Introduction:**

Fecal incontinence (FI) is a prevalent yet underreported condition that significantly impairs quality of life and social functioning. Conservative treatment, particularly pelvic floor muscle training (PFMT), plays a key role in patients with mild sphincter dysfunction or those not eligible for surgical intervention. However, the impact of professional supervision on the effectiveness of PFMT remains insufficiently explored.

**Methods:**

The study included 124 patients with fecal incontinence treated at a proctology department and 30 patients in the study control group (without fecal incontinence). The study group was divided into two groups: patients performing PFMT under physiotherapist supervision (*n* = 64) and patients exercising independently without professional supervision (*n* = 60). All participants underwent anorectal manometry and completed questionnaires assessing fecal incontinence severity using a modified Wexner scale before and after a 6-week exercise program. Resting and squeeze anal pressures were analyzed.

**Results:**

Before therapy, no statistically significant differences were observed between the groups. After completion of the rehabilitation program, patients exercising under physiotherapist supervision showed a significantly greater reduction in fecal incontinence-related lifestyle alterations than the unsupervised group. Supervised PFMT was associated with a significant improvement in functional outcomes, whereas no significant improvement was observed in patients exercising independently. In both groups, an increase in sphincter contraction pressure and improved retention of liquid stool were noted.

**Conclusions:**

Pelvic floor muscle exercises are an effective conservative treatment for fecal incontinence; however, physiotherapist-supervised training yields superior clinical outcomes compared with unsupervised exercise. Professional supervision, appropriate patient qualification, and patient motivation are key factors influencing the success of pelvic floor rehabilitation.

## Introduction

Fecal incontinence (FI) is a common issue, but many patients feel embarrassed to seek medical help. Approximately 50% of FI is a result of the leakage of liquid stool through fecal masses in people with chronic constipation. Long-lasting fecal mass retention results in excessive stretching of the anorectal muscles and a change in the anorectal angle, both of which lead to patients’ loss of ability to distinguish liquid stool, solid stool, and gas [1].

Although FI occurs in both sexes, women are more frequently affected by the condition. This is due to the female pelvic structure, hormonal influences, and prior childbirth. Giving birth is the most important causative factor of sphincter insufficiency. Even physiological childbirth can be a reason for fecal and gas incontinence in 30% of women. Among many other reasons for FI, hemorrhoids, prolapsed rectum, fistulas, infectious diseases, and neuropathies can be mentioned [2].

The problem of FI is significant not only for hygienic reasons but also for sociological reasons. The lack of ability to defecate is a kind of disability that results in the exclusion of the patient from family and social life, limiting such activities [3].

Idiopathic dysfunction of the sphincter apparatus can be treated surgically or conservatively. Conservative treatment can be used in patients with mild sphincter defects or in those who have decided not to undergo surgery.

Physiotherapeutic methods of conservative treatment are most significant and include, among others, pelvic floor muscle exercises (PFMT). PFMT is a structured program of voluntary, repetitive contractions and relaxations of the pelvic floor muscles aimed at improving the strength, endurance, coordination, and functional support of the pelvic organs, thereby enhancing continence and pelvic stability [4].

## Methods

The study group consisted of 124 patients treated in the Outpatient Proctology Clinic at Poznań University of Medical Sciences. There were 108 women and 16 men, divided into two groups. Additionally, 30 patients without fecal incontinence were included in the study’s control group.

The exclusion criteria were: a history of natural childbirth, injuries or surgeries of the pelvic floor, and surgical or proctological treatment for conditions such as hemorrhoids, anal fissures, and rectal prolapse.

The first study group encompassed patients performing pelvic floor exercises under the supervision of a physiotherapist (*n* = 64). Most of the individuals were women (55 patients, 85.9%) and people aged 60–79 years (24 patients, 37.5%).

The second group (*n* = 60) included patients performing pelvic floor exercises without professional supervision. The group was dominated by women (50 patients, 83.3%) and by people over 80 years (26 patients, 43.4%).

The control group included patients without fecal incontinence or other proctologic diseases. The group consisted of 25 women and 5 men, with a mean age of 62 years.

Most patients in both study groups had primary education (47.58%). In terms of occupation, most respondents worked in white-collar jobs (29.83%) or were unemployed at the time of the study (28.22%).

The characteristics of the study group are presented in the table below (Table 1).

**Table 1:** Study group characteristics

Features	Patients performing pelvic floor exercises without professional supervision *N* = 60	Patients performing pelvic floor exercises under the supervision of a physiotherapist *N* = 64	Control group *N* = 30
Gender			
Female	50	55	25
Male	10	9	5
BMI			
< 25 kg/m^2^	17	19	15
25–30 kg/m^2^	25	30	8
> 30 kg/m^2^	18	16	7
Age, years			
19–39	4	2	15
40–59	12	16	10
60–79	24	20	5
> 80	20	26	0
Education			
Primary	31	28	8
Vocational	10	9	7
Secondary	10	15	7
Higher	9	12	8
Type of work/lifestyle			
Mental	18	19	18
Sedentary physical	12	15	5
Motion physical	14	11	7
Nonworking (pensioner)	16	19	0

*BMI* body mass index

To assess the severity of fecal incontinence across all patients, a modified Wexner scale was used. The study included anorectal manometry before and after the rehabilitation program. The manometric examination was performed in an outpatient proctology clinic. An eight-channel, balloon-tipped flow probe was used. Data were recorded using the Polygraf HR PC system, which is used for stationary examinations of the upper and lower gastrointestinal tracts. Data analysis was performed using Synectics Medical Polygram software. The control group was examined only once to assess and validate a manometry method.

During the examination, the following were assessed: resting and sphincter maximal pressures, duration of sustained squeeze, anal squeeze increment, residual anal pressure, and rectal pressure.

In the first meeting with the physiotherapist, each patient received detailed instructions on how to perform pelvic floor exercises and a set of ten exercises used and discussed during therapy to be performed at home. The exercises were ranked by level of difficulty, allowing gradual progression from lying down to all fours to standing. The patient developed this set on the basis of strictly defined principles and available substantive sources Table 2.

**Table 2:** Wexner incontinence scoring scale

Type of incontinence	Frequency				
	Never	Rarely	Sometimes	Usually	Always
Solid	0	1	2	3	4
Liquid	0	1	2	3	4
Gas	0	1	2	3	4
Wears pad	0	1	2	3	4
Alteration in lifestyle	0	1	2	3	4

Never: 0; rarely: < 1/month; sometimes: < 1/week, > 1/month; usually: < 1/day, > 1/week; always: > 1/day, 20: complete incontinence

After a 6-week period (during which the patients performed the recommended pelvic floor exercises), a repeat manometric examination and a second survey were conducted. Patients in the first group were scheduled for a follow-up appointment with the physiotherapist. The purpose of the second anorectal manometry was to assess the functional status of the anal sphincter muscle structures following physiotherapy. During the second meeting with the patients, the physiotherapist assessed the accuracy and correctness of the exercise performance and recorded any comments.

Shapiro–Wilk and Kolmogorov–Smirnov tests were conducted to assess the normality assumption (*p*-value above 0.05). Differences between groups 1 and 2 were analyzed using the Mann–Whitney *U* test (when both groups had a normal distribution), Student’s *t*-test (when both groups did not have a normal distribution), or the chi-squared test for categorical variables. Statistically significant results were reported when the *p*-value was less than 0.05. All analyses were performed using StatSoft Statistica version 14.0.

## Results

Mean resting pressure and mean maximum pressure differed significantly between the study groups and the control group (*p* < 0.005). Differences were also observed in other parameters measured using dynamic transrectal manometry. However, no statistically significant differences were observed in transrectal manometry measurements between the groups of patients with fecal incontinence. These results are presented in Table 3.

**Table 3:** Initial manometry results in the control and study groups

Features	PFMT without professional supervision	PFMT under the supervision of a physiotherapist	Control group
Anal resting pressure (mean ± SD)	14.76 ± 1.46 mmHg	13.67 ± 1.98 mmHg	63.23 ± 4.8 mmHg
Anal squeeze pressure (mean ± SD)	97.76 ± 5.64 mmHg	92.23 ± 6.79 mmHg	143.56 ± 10.98 mmHg
Duration of sustained squeeze (mean ± SD)	11.56 ± 4.6 s	15.67 ± 3.43 s	35.30 ± 5.62 s
Anal squeeze increment (mean ± SD)	110.87 ± 12.89 mmHg	105.45 ± 8.76 mmHg	155.87 ± 23.12 mmHg
Residual anal pressure (mean ± SD)	67.89 ± 8.45 mmHg	74.56 ± 10.23 mmHg	92.76 ± 8.90 mmHg

*SD* standard deviation

Baseline results for the features assessed by manometry showed no statistically significant difference between the study groups. All measurements were significantly lower than those in the control group and indicated anal sphincter insufficiency across all parameters. Measurements in the control group were within the normal range.

There were no statistically significant differences in the final score or the individual components of the Wexner scale between the two study groups. Most patients reported liquid fecal and gas incontinence, which occurred at least once a week, and in many cases, daily. Over 80% of patients also reported the need for lifestyle changes owing to fecal incontinence symptoms and considered this to be a constant occurrence. Only less than 30% of patients rated solid fecal incontinence as occurring more than once a week. Approximately 35% of patients reported the need to wear an incontinence pad daily or more than once a week. Results are presented in Table 4.

**Table 4:** Assessment of Wexner scale and particular features in the study groups before start of treatment with or without a physiotherapist

Features	PFMT without professional supervision	PFMT under the supervision of a physiotherapist	*p*-Value
Wexner scale score (median)	12	14	0.56
Solid stool incontinence (median)	2	2	0.43
Liquid stool incontinence (median)	3	3	0.87
Gas incontinence (median)	3	2	0.67
Wears pad (median)	2	2	0.43
Alteration in lifestyle (median)	2	3	0.23

It should be noted that the mean resting pressures in the study groups after PMRT were still lower than expected norms, but they increased significantly compared with the results before PFMT. Resting pressure was also significantly higher, favoring patients who performed PFMT under the supervision of a physiotherapist (*p* = 0.00043).

Significant improvement was also observed in the assessment of maximum anal canal pressure, and pressures increased during follow-up manometry. The group performing regular exercises under the supervision of a physiotherapist achieved statistically significantly better results than the group performing unsupervised exercises (*p* = 0.0076).

Other manometric parameters also improved significantly, and statistically significant correlations were observed. A summary of the results is presented in Table 5.

**Table 5:** Results and comparative assessment of key parameters obtained from anorectal manometry, including the Werner scale and an in-depth analysis of its extended subcomponents after PFMT

Features	Exercises without professional supervision	Exercises under the supervision of a physiotherapist	*p*-Value
Anal resting pressure (mean ± SD)	20.54 ± 2.56 mmHg	25,32 ± 3.44 mmHg	0.00043
Anal squeeze pressure (mean ± SD)	115.26 ± 8.67 mmHg	123.98 ± 11.23 mmHg	0.0076
Duration of sustained squeeze (mean ± SD)	23.16 ± 9.6 s	27.98 ± 8.98 s	0.0041
Anal squeeze increment (mean ± SD)	134.17 ± 12.89 mmHg	147.95 ± 14.26 mmHg	0.024
Residual anal pressure (mean ± SD)	82.12 ± 10.42 mmHg	88.16 ± 8.83 mmHg	0.0089
Wexner scale score (median)	8	4	0.0032
Solid stool incontinence (median)	2	1	0.012
Liquid stool incontinence (median)	2	1	0.053
Gas incontinence (median)	2	1	0.069
Wears pad (median)	1	1	0.067
Alteration in lifestyle (median)	2	1	0.067

The above analyses indicate a significant improvement in Wexner scale scores in both study groups compared with the pre-PFMT assessment. Additionally, statistically significant differences were observed between the study groups in Wexner scale scores, favoring the group performing exercises under the supervision of a physiotherapist (*p* = 0.0032). Statistically significant improvements were also observed in incontinence of solids, liquids, and gases when comparing pre- and post-PFMT conditions. However, no significant differences were found between the study groups in improvement in liquid and gas incontinence, or in lifestyle changes. The group performing exercises under the supervision of a physiotherapist had significantly fewer incontinence pad changes in the final results.

Additionally, the Wexner scale was highest among patients with primary education and significantly higher than among those with higher education (*p* = 0.0028). However, patients with physical motion work and those who did not perform any work (pensioner) had significantly higher Wexner scale scores than patients with mental work (*p* = 0.045 and 0.0034, respectively). The results are presented in Table 6.

**Table 6:** The Wexner scale score results depending on the type of work/lifestyle or education

Features/*p*-value	Primary	Vocational	Secondary	Higher
Primary	−	0.078	0.098	0.0028
Vocational	0.078	−	0.78	0.059
Secondary	0.098	0.78	−	0.062
Higher	0.0028	0.059	0.062	−
Features/*p*-value	Mental	Sedentary physical	Motion physical	Nonworking (pensioner)
Mental	−	0.067	0.045	0.0034
Sedentary physical	0.067	−	0.076	0.089
Motion physical	0.045	0.076	−	0.055
Nonworking (pensioner)	0.0034	0.089	0.055	−

## Discussion

FI is a common and socially disabling condition. Although it greatly affects quality of life, FI is often underdiagnosed and undertreated, with conservative treatments not used as often as they should be. This prospective study shows that PFMT significantly enhances anorectal manometric results and reduces symptoms in patients with idiopathic sphincter dysfunction. Notably, patients who received supervised physiotherapy achieved much better outcomes than those who performed unsupervised home-based exercises.

The observed increases in resting and squeeze anal pressures confirm that PFMT strengthens both the tonic and phasic aspects of sphincter function. However, evidence from recent studies shows that the benefits of PFMT go beyond just muscle hypertrophy. Neurophysiological research suggests that repeated pelvic floor exercises enhance rectoanal sensory integration, cortical motor control, and reflex coordination, helping restore continence mechanisms even when the sphincter complex’s anatomy is not fully intact [5, 6]. This multifaceted effect likely explains the significant reduction in symptoms observed in our group, despite post-treatment pressures remaining below normal values.

A key discovery of this research is that patients under professional supervision experience notably better therapeutic responses. Supervised pelvic floor muscle training (PFMT) enables accurate muscle engagement, real-time adjustments to compensate for improper technique, a gradual increase in resistance, and continuous patient motivation. These elements are consistently linked to improved outcomes in pelvic floor therapy [7]. Studies comparing supervised PFMT with education-only or self-guided programs show that structured physiotherapy more effectively reduces episodes of fecal incontinence, improves continence scores, and enhances quality of life [8, 9]. Our data support these conclusions, with supervised groups showing higher manometric pressures, prolonged squeeze durations, and lower Wexner scores.

Both liquid stool and gas incontinence improved across all study groups, indicating that PFMT likely enhances anorectal sensory discrimination and reflex responses, not just mechanical closure pressure. Sensory dysfunction is increasingly seen as a key factor in FI, especially in idiopathic and post-obstetric cases [10]. The improvements in this study probably result from restoring coordinated rectal sensation and sphincter response via neuromuscular retraining.

From a clinical management standpoint, these results support the growing view that conservative therapy should be the first-line treatment for FI. Surgical options such as sphincteroplasty, sacral neuromodulation, and injectable bulking agents may help certain refractory cases, but their long-term success varies, with decreasing continence and high reintervention rates [11]. Today, international guidelines favor structured pelvic floor rehabilitation as the primary approach, with invasive procedures reserved for patients who do not respond to optimized conservative methods [12, 13].

Adherence is crucial for therapeutic success. Supervised programs generally lead to better compliance, greater motivation, and more precise exercise execution than unsupervised programs [14]. The improved results in our supervised group probably stem from both physiological benefits and behavioral reinforcement, highlighting the importance of collaboration between physicians and physiotherapists.

In pelvic floor rehabilitation, the therapist not only monitors squeezing strength but also identifies and corrects abnormal muscle recruitment patterns. Patients with FI often exhibit dyssynergic activation patterns, which, if not recognized and addressed during training, can considerably reduce treatment effectiveness [15].

### Limitations

Several limitations must be recognized. The follow-up period was relatively short, limiting the ability to evaluate the long-term durability of PFMT benefits. Variations in age and educational background may have affected adherence and learning capacity.

Future research should compare fully home-based treatments, supervised outpatient rehab, and hybrid approaches. It should also evaluate muscle recruitment quality and look for compensatory activation patterns during therapy.

Additionally, studies could enhance patient stratification by incorporating methods such as biofeedback or imaging-based sphincter assessment.

## Conclusions

In all patients, both when exercising alone and under the supervision of a physiotherapist, there was an increase in sphincter contraction pressure and improved stool retention, indicating that pelvic floor muscle exercises are practical for treating fecal incontinence.

The main clinical parameters influencing the effectiveness of sphincter rehabilitation through exercises are intrinsic motivation, professional supervision, and proper physician qualification.

The efficacy of exercises supervised by a physiotherapist is higher than that of exercises performed independently by the patient.

The suggested home-based protocol could be a valuable addition to an organized rehabilitation program, especially after an initial supervised assessment and training session with an experienced pelvic floor muscle therapist. However, we should avoid suggesting that home biofeedback can fully replace supervised pelvic floor muscle rehabilitation.

## Data Availability

No datasets were generated or analyzed during the current study.
